# The Effect of Rhodamine-Derived Superparamagnetic Maghemite Nanoparticles on the Motility of Human Mesenchymal Stem Cells and Mouse Embryonic Fibroblast Cells

**DOI:** 10.3390/molecules24071192

**Published:** 2019-03-27

**Authors:** Larisa Baiazitova, Josef Skopalik, Jiri Chmelik, Inna Zumberg, Vratislav Cmiel, Katerina Polakova, Ivo Provaznik

**Affiliations:** 1 Department of Biomedical Engineering, Faculty of Electrical Engineering and Communication, Brno University of Technology, Technicka 3082/12, 61600 Brno, Czech Republic; xbaiaz00@stud.feec.vutbr.cz (L.B.); skopalik@feec.vutbr.cz (J.S.); chmelikj@feec.vutbr.cz (J.C.); xzumbe00@stud.feec.vutbr.cz (I.Z.); cmiel@feec.vutbr.cz (V.C.); 2 Regional Centre of Advanced Technologies and Materials, Faculty of Science, Palacky University, 17 listopadu 12, 771 46 Olomouc, Czech Republic; dr.kacka.polakova@gmail.com

**Keywords:** magnetic nanoparticles, mesenchymal stem cells, fibroblast cells, cytotoxicity, wound healing assay, single-cell migration

## Abstract

Nanoparticles have become popular in life sciences in the last few years. They have been produced in many variants and have recently been used in both biological experiments and in clinical applications. Due to concerns over nanomaterial risks, there has been a dramatic increase in investigations focused on safety research. The aim of this paper is to present the advanced testing of rhodamine-derived superparamagnetic maghemite nanoparticles (SAMN-R), which are used for their nontoxicity, biocompatibility, biodegradability, and magnetic properties. Recent results were expanded upon from the basic cytotoxic tests to evaluate cell proliferation and migration potential. Two cell types were used for the cell proliferation and tracking study: mouse embryonic fibroblast cells (3T3) and human mesenchymal stem cells (hMSCs). Advanced microscopic methods allowed for the precise quantification of the function of both cell types. This study has demonstrated that a dose of nanoparticles lower than 20 µg·cm^−2^ per area of the dish does not negatively affect the cells’ morphology, migration, cytoskeletal function, proliferation, potential for wound healing, and single-cell migration in comparison to standard CellTracker™ Green CMFDA (5-chloromethylfluorescein diacetate). A higher dose of nanoparticles could be a potential risk for cytoskeletal folding and detachment of the cells from the solid extracellular matrix.

## 1. Introduction

Recently, nanoparticles have attracted the interest of scientists in various areas of biomedical research because of their unique properties. Nanoparticles of iron oxide, such as Fe_3_O_4_ and γ-Fe_2_O_3,_ have been reported to be applicable as a material for use in drug delivery systems, magnetic resonance imaging [1], cancer therapy, and other fields. A potentially promising member of the family of iron oxides, maghemite (Fe(II)-deficient magnetite), allows the manufacture of nanoparticles that are biocompatible and non-toxic to living organisms. Maghemite nanoparticles can be introduced to living cells and their magnetic properties allow remote manipulation with external magnetic fields [2,3]; specific separation of cells from blood or mixed cell culture; inducing hyperthermia for selective destruction of cancer cells [4]; and delivery of pharmacoactive compounds or genes, and their selective activation by external stimuli [5]. Recently, cell labelling by superparamagnetic iron oxygen (SPIO) nanoparticles has also been used for cell tracking in vitro or in vivo. Multimodal probes based on SPIO nanoparticles may serve for cell detection and cell tracking via complementary contrast in light absorption or fluorescence. SPIO nanoparticles have also attracted much consideration for the possibility of replacing cytosolic or membrane probes, which are used in fluorescent confocal microscopy and microscopic time-lapse experiments due to their long-term chemical stability and photostability [6].

Rhodamine-derived superparamagnetic maghemite nanoparticles (SAMN-R) display relatively high uptake by mammalian cells, very good stability in intracellular spaces, and long-term stability of their rhodamine fluorescence shell in intracellular space with a specific pH. SAMN-R have been previously tested on rat, rabbit and human stem cells, and are presented as a robust cell marker, a long-term stable probe for deposition in lysosomes, a probe that does not affect nuclei, and a probe with theranostic potential [7,8].

The main aim of this article is to expand on cell labelling experiments and refocus from the basic tests of acute toxicity and contrast properties to advanced evaluation of their interactions with mesenchymal stromal cells (MSCs) and fibroblasts, as well as evaluating their influence on the migration potential of cells. Fibroblasts and MSCs play a key role in regenerative medicine, especially in the regeneration of the dermis, chondral intercostal muscles, and other tissues for their motility and migration ability [9,10,11]. Thus, further investigation of SAMN-R properties will potentially bring new insight into the possibility of future biomedical applications.

## 2. Results

### 2.1. Cell Morphology and Flow Cytometry

**Figure 1:**
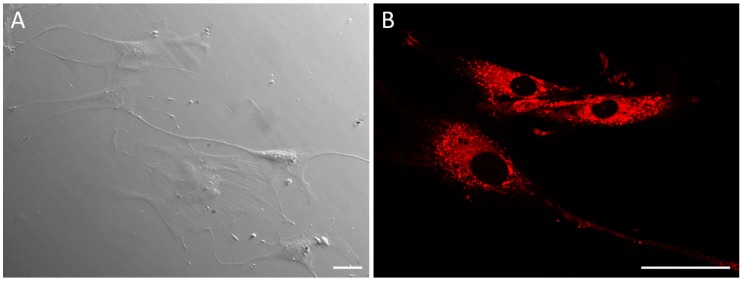
Human mesenchymal stem cells (hMSCs) treated with rhodamine-derived superparamagnetic maghemite nanoparticles (SAMN-R). (**A**) Integrated modulation contrast in wide-field microscopy, 40× magnification; (**B**) confocal microscopy, 63× magnification. Scale bar 50 µm.

SAMN-R have been studied previously [7,8,12]. The stability of SAMN-R and their maghemite structure was confirmed by Mössbauer spectroscopy in the first days after preparation and also one year after preparation. The polydisperse index was quantified in all experiments using dynamic light scattering, its value being 0.22 ± 0.03 in dH_2_O and 0.47 ± 0.03 in culture medium. Their size varied from 20 to 50 nm [12]. It was found that nanoparticles are localized in lysosomes surrounding stem cell nuclei and are not localized in the cell nuclei themselves [8] (see Figure 1).

In this study, a 50 µg·mL^−1^ concentration of SAMN-R was used. Currently, there are many different types of dishes, well plates, or cultivation chambers, and labelling with SAMN-R has to be optimized for use in various types of cultivation chambers. However, SAMN-R are not uniformly distributed in the labelling suspension.

The nanoparticles settle on the surface of cells and culture dishes due to agglomeration and gravity in the process of labelling. It should be noted that the optimal concentration may be close to the toxicity limit in samples with a small surface area. Therefore, we used dose per surface area rather than concentration as a volume in our study.

**Figure 2:**
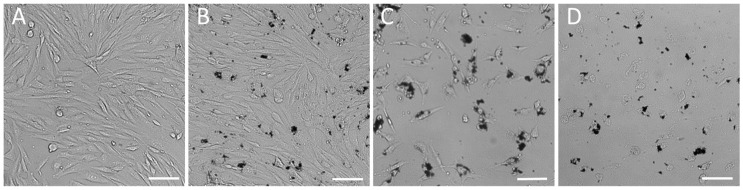
Influence of SAMN-R in different doses to mouse embryonic fibroblast cells (3T3) morphology. (**A**) Control; (**B**) dose of SAMN-R 20 µg·cm^−2^; (**C**) dose of SAMN-R 25 µg·cm^−2^; (**D**) dose of SAMN-R 35 µg·cm^−2^. Bright-field microscopy, 10× magnification, scale bar 100 µm.

In our research, we used fibroblast cell types—large size human mesenchymal stem cells (hMSCs) (about 250 μm) and considerably smaller mouse embryonic fibroblast cells (3T3) (about 90 μm). At the dose of SAMN-R under 20 µg·cm^−2^, visible changes in cell shape and membrane integrity were not observed (Figure 2A,B). When the dose increased to more than 20 µg·cm^−2^, we noticed a decline in cell proliferation ability and the appearance of necrotic and detached cells (Figure 2C,D).

We found out that even at the lowest tested doses, the SAMN-R emitted enough fluorescence to be detectable. According to the results of this experiment, it was decided to test the dose of SAMN-R of 20 µg·cm^−2^, since it was determined as the limiting value.

**Figure 3:**
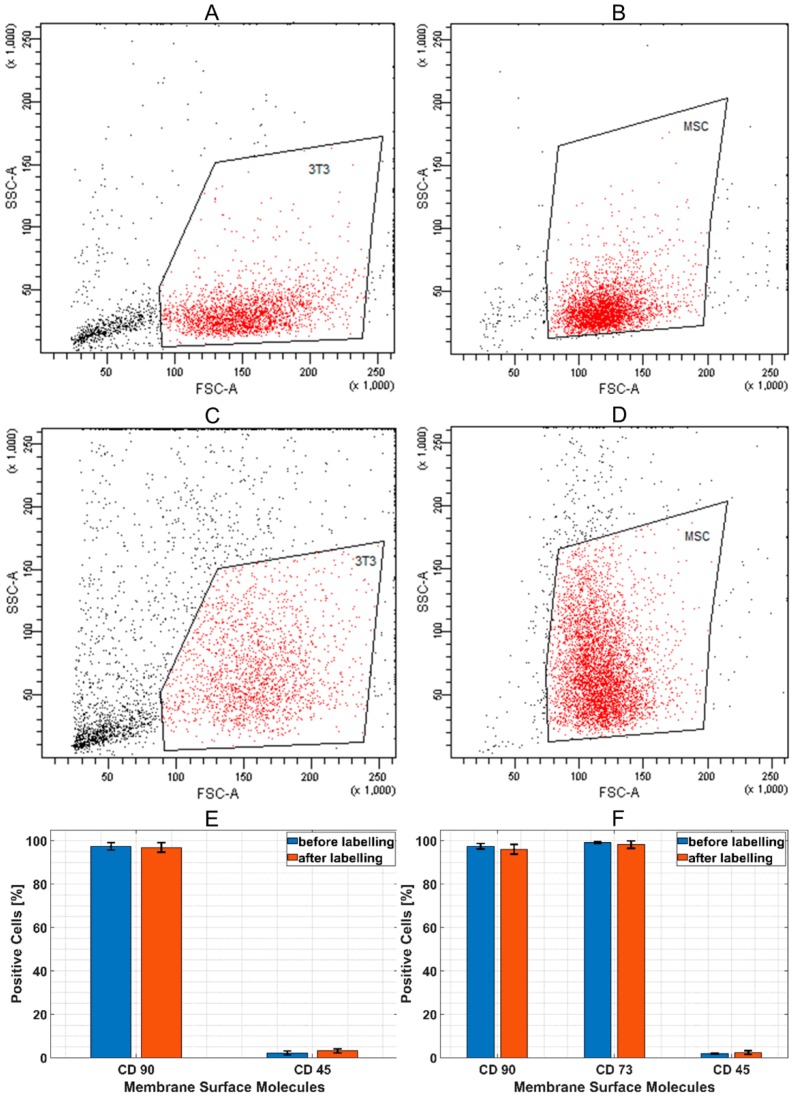
Flow cytometry analysis of labelled cells. Site scatter and forward scatter: (**A**) 3T3 before labelling; (**B**) mesenchymal stromal cell (MSC) before labelling; (**C**) 3T3 after 24 h of labelling; (**D**)MSC after 24 h of labelling; (**E**) quantification of CD90 and CD45 positive 3T3; (**F**) quantification of CD90, CD73, and CD45 positive MSC.

Flow cytometry evaluation gives a statistical comparison of cell size and cell granularity before and after nanoparticle incorporation. Granularity of both cell types was increased after nanoparticle deposition in intracellular space. However, size and distribution of cluster of differentiation (CD) molecules on the cell surface were not affected (Figure 3).

### 2.2. Quantification of Reactive Oxygen Species (ROS) Generation after SAMN-R Labelling

**Figure 4:**
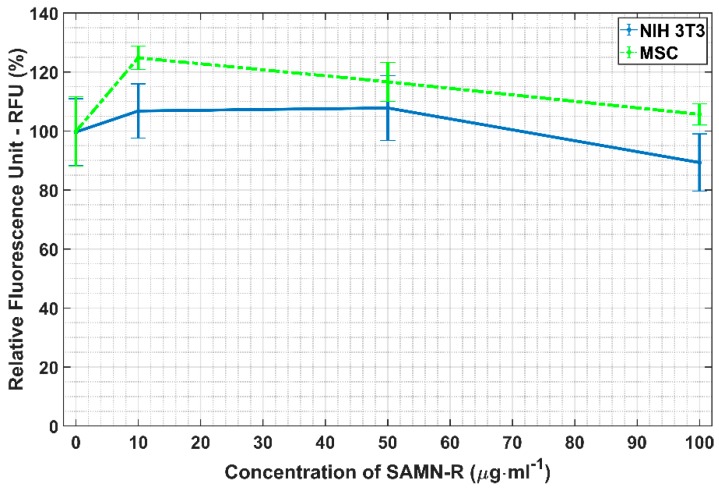
Measurement of reactive oxygen species (ROS) after cell labelling. The intensity of indicator CM-H2DCFDA was measured for four different labelling concentrations. The horizontal segment (whiskers) marks the standard deviation of fluorescence intensity.

The value of ROS production is measured by CM-H2DCFDA after the exposure of cells to SAMN-R, which is an aspect that helps to quantify the very early changes in cell physiology. This method is very sensitive in many cases of cell stress, very often for several hours before the start of any apoptosis or cell malformation. The CM-H2DCFDA kit determines the rate of production of hydrogen peroxide (H_2_O_2_), hydroxyl radicals (^•^HO), and superoxides (O_2_^−^) in the cells. The final graphical overview of ROS value measured on our cells after SAMN-R labelling is shown in Figure 4. The results show that almost all used labelling concentrations do not have any negative effects on ROS value.

### 2.3. Cell Proliferation

**Figure 5:**
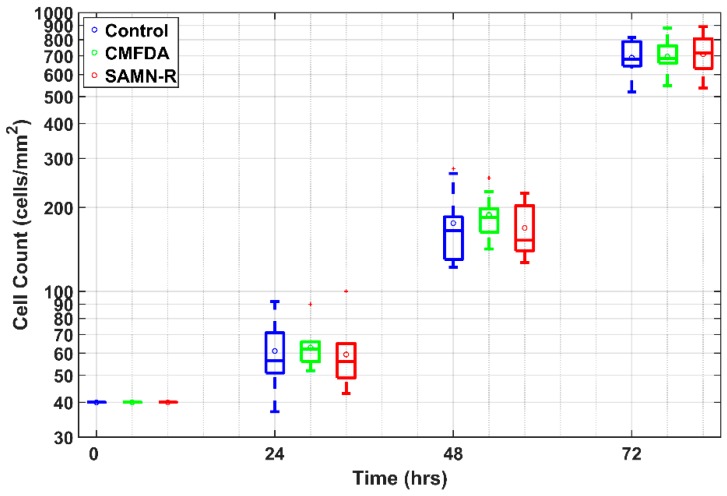
3T3 cell growth. Each box represents the number of cells in an area of 1 mm^2^ from 10 experiments. Each box plots the 25th and 75th percentiles. The circle inside the box is the mean, the band inside the box is the median. Whiskers (the most extreme values not considered as outliers) and outliers (red squares) are outside the box.

A fluorescence probe designed for live cell imaging can be toxic for cells [13,14]. Negative effects on vital functions could otherwise affect the positive properties and application potential of the dye. As an initial study, we used a cell growth curve to identify the effect on cell proliferation (Figure 5).

For comparison with SAMN-R, we tested CellTracker™ Green CMFDA (5-chloromethylfluorescein diacetate) because it is a low toxic reagent. The result of the statistical *t*-test showed that both dyes do not have a significant effect on cell proliferation. This means that SAMN-R can be successfully used in long-term experiments such as a cell tracking dye similar to CMFDA.

### 2.4. Cell Migration Study

Currently, researchers use many methods to study cell motility such as wound healing assays, trans-well cell migration, cell exclusion zone assays, spheroid migration assays, single-cell motility assays, and others [15]. Wound healing assays, a widely used method for the analysis of cell migration, are treated with a specific compound [16,17,18]. This method is technically non-demanding, and it is possible to observe changes in cell motility in a short time [19]. To understand the effect of changes in the environment on the mechanical responses of cells, single-cell migration was usually used in other studies [20,21].

#### 2.4.1. In Vitro Wound Healing Assay

**Figure 6:**
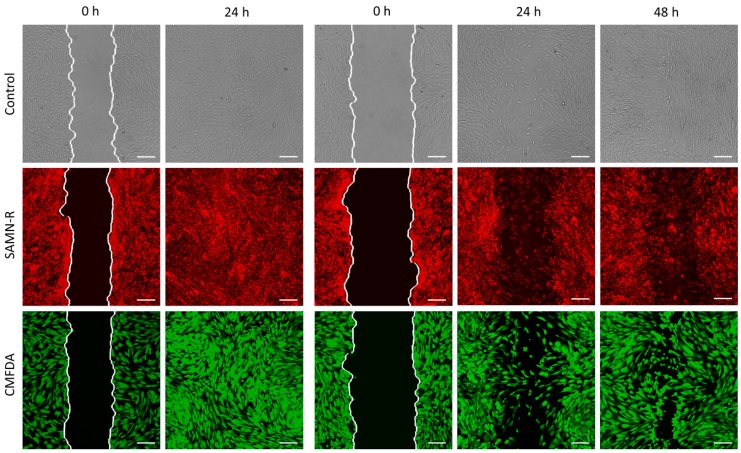
Wound healing assay applied to 3T3 cells: control, SAMN-R, and CellTracker™ Green CMFDA (5-chloromethylfluorescein diacetate) labelled cells. Images were detected at the beginning and after 24 and 48 h. The white line marks the initial scratch border. Confocal microscopy, 10× magnification, scale bar 150 µm.

In each experiment, three groups of 3T3 cells were investigated: control (non-labelled), treated SAMN-R, and those labelled with CMFDA dye. We studied the effect of these treatments on the ability of the collective migration of 3T3 cells by wound healing assays. In 10 experiments, we obtained 60 images from each group: 30 with a narrow scratch and 30 with a wide scratch, which were compared separately (Figure 6).

**Figure 7:**
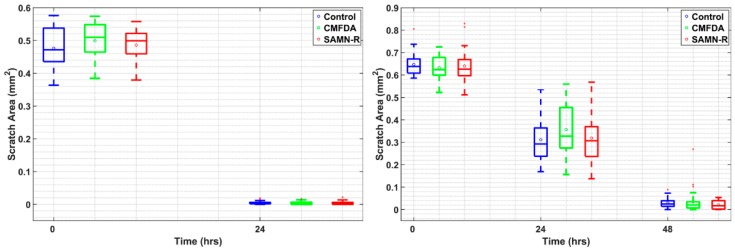
Time-dependent changes in the size of the open area due to 3T3 cell migration (**left**) in narrow scratch and (**right**) in wide scratch. Each box represents scratch areas in 30 images in each group. Each box plots the 25th and 75th percentiles. The circle inside the box is the mean, the band inside the box is the median. Whiskers (the most extreme values not considered as outliers) and outliers (red squares) are outside the box.

Since the cells migrate as a loosely connected population, the analysis was based on calculating the clear area sites present at the wound. Quantitative analysis was provided using a custom-made algorithm in MATLAB software (R2018a 64-bit, MathWorks®, Natick, MA, USA). As can be seen from the data in Table 1 and Table 2, the areas with a narrow scratch were overgrown within approximately 24 h, and the areas with a wide scratch were overgrown within 48 h. Time-dependent changes in the open area due to 3T3 cell migration are shown in Figure 7.

A statistical *t*-test shows that the effect of SAMN-R and CMFDA dye to collective cell migration is not statistically significant.

#### 2.4.2. Single-Cell Migration

To investigate single-cell migration, we used a time-lapse microscopy scan to observe hMSCs. The selected scan field was scanned over six hours at intervals of five minutes. In the primary experiments (Patient 1), there were scans only in one field of view. The following experiments were made with a choice of two to four fields (Patients 2–4) with the help of multi-view mode (mark and find function). The calculated value of velocity and accumulated and Euclidean distance hMSCs are listed in Table 3, Table 4, Table 5 and Table 6.

**Figure 8:**
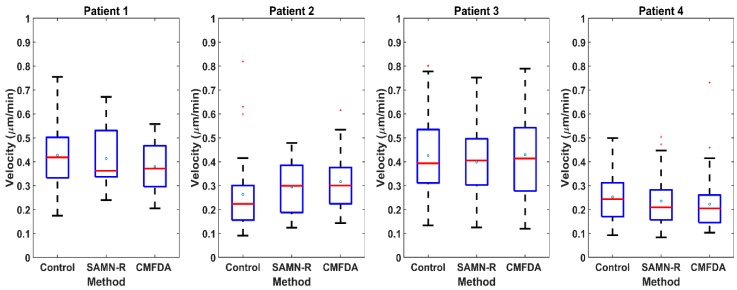
Calculated velocity from single hMSCs. Each box represents the complete set of single-cell velocities from four patients. There are 18 cells in each group from Patient 1, 30 cells from Patient 2, and 50 cells from Patients 3 and 4. Boxes are composed of main box edges (25th and 75th percentiles), blue dots (mean), central red horizontal line (median), whiskers (the most extreme values not considered outliers), and red squares (outliers).

**Figure 9:**
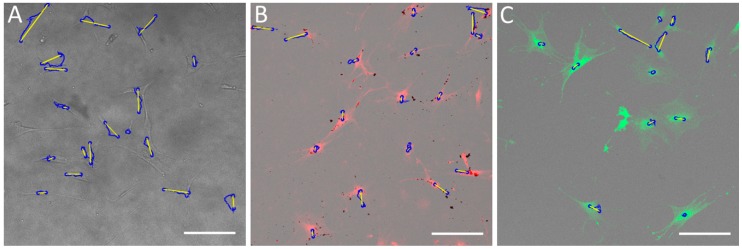
Tracks of hMSCs. (**A**) Non-labelled cells (control); (**B**) SAMN-R treated cells; (**C**) CMFDA-labelled cells. The blue circle is a detected cell, the blue line is accumulated distance, and the yellow line is Euclidean distance. Confocal microscopy, 10× magnification, scale bar 250 µm.

In our research, the most important parameter during cell migration of single cells is velocity. Figure 8 presents the complete set of single-cell velocities from four patients. The number of cells in groups from one patient was the same. Using a statistical *t*-test, it was shown that the effect of SAMN-R and CMFDA dye on individual hMSCs’ velocity was not statistically significant. The Rayleigh test confirms that cell distribution is homogeneous. Cell tracking examples are shown in Figure 9.

## 3. Discussion

Fibroblasts and mesenchymal stem cells have prominent status in the hierarchy of mammal cells. Fibroblasts are one of the most populous and important cells of connective tissue, and play the crucial role in normal pre- and postnatal organ development. They represent key components for wound healing and the occurrence of tumors. MSCs are relatively more latent cells in normal development; however, their effective migration, proliferation, and interaction with other cell types are essential in the critical time after tissue scarring or ischemic events, when wound healing and correction of abnormal immune reactions begins. Unfortunately, fibroblasts and MSCs could be negatively affected by modern cell markers, cell labels, or labelling nanoparticles [22,23].

The negative effect of markers and nanoparticles may occur a short time after their application. Such markers and nanoparticles with acute toxicity cause visible rapid morphological changes and cells rapidly die. Markers and nanoparticles, that can be seemingly non-toxic, may have a hidden long-term negative effect that can manifest after several days. Moreover, the effect need not necessarily lead to apoptosis of the cell, but rather to the abnormal production of certain factors in the cell (e.g., reactive oxygen) or to altered metabolism or cell motility.

We have focused on the evaluation of the abovementioned hidden long-term effects after the application of SAMN-R to cells. Acute toxicity and critical concentration/dose for immediate effective marking of cells were described in our previous studies [7,8].

In the majority of publications dealing with the toxicity of nanoparticles, the authors take as a standard measure the absolute concentration of the nanoparticles in a given cultivation medium presuming colloidal stability of nanoparticle suspension. However, there is a certain agglomeration rate and sedimentation rate dependent on particles’ concentration even in case of almost ideally colloidal particle suspensions [24]. With regard to increasing concerns about the health risks of nanoparticles, we propose to define concentrations of each used solution in µg·mL^−1^ with a value of a dose in µg·cm^−2^ per area of the dish with medium, although an increase of particles’ concentration in the microenvironment of cells does not exceed 10% (data not shown).

The first set of experiments included cultivation of fibroblasts at the bottom of standard in vitro dishes treated with SAMN-R. The used SAMN-R treatment solutions were prepared in a gradient series. Nanoparticles were diluted in a cultivation medium, and a colloid profile of solution was prepared by 20 min of ultrasound exposure immediately before cell incubation. The used absolute concentrations were 0, 10, 20, 30, 40, 50, 60, and 70 µg·mL^−1^, which corresponded to doses of 0, 5, 10, 15, 20, 25, 30, and 35 µg·cm^−2^ per area of the dish. The effects of different doses of SAMN-R were evaluated after 24 h of incubation. Cell cultures with a dose of SAMN-R up to 20 µg·cm^−2^ did not display a difference in comparison to control cells. These doses of SAMN-R under 20 µg·cm^−2^ did not induce visible changes in cell shape and membrane integrity (see Figure 2A,B). With increasing doses above 20 µg·cm^−2^, we noticed a decline of cell proliferation ability and the appearance of necrotic and detached cells (see Figure 2C,D). Flow cytometry analysis showed that cell morphology and cell size were not changed for this SAMN-R concentration range. Another cell culture up to 20 µg·cm^−2^ SAMN-R dose displayed cell detachment or inhibited cell growth. The value 20 μg·cm^−2^ was also detected as being critical for 3T3 and MSCs; the ratio of events of cell detachment and inhibition of cell growth was similar.

Cells utilize two main migration modes in mammals: single-cell migration and collective migration. The single-cell migration is characteristic for immune cells or MSCs after some injuries. Collective migration is particularly important in tissue shaping and wound healing, such as epithelial regeneration or fibroblast closure of wounds. Both modes can be affected by present nanoparticles, and several previously published articles warn of some types of nanoparticles which could potentially induce negative affecting of these processes [25,26,27,28].

Our time-lapse microscopical visualization of MSCs’; motility and its statistical summary in MATLAB give clear conclusions: SAMN-R does not affect typical hMSCs’ ameboid motility, nor does it affect the average velocity of the cells. The used dose of SAMN-R of 20 µg·cm^−2^ displayed high biocompatibility and no effect on cell motility, which is comparable with the CMFDA commercial cell tracker marker (see Figure 8 and Table 3, Table 4, Table 5 and Table 6). Further motility tests performed on fibroblasts also gave positive results for SAMN-R. Covering of scratches in a fibroblast monolayer labelled by SAMN-R was not affected compared to non-labelled fibroblast experiments. Migration of SAMN-R labelled cells was also compared with migration of cells labelled by commercial low toxicity CMFDA cell tracker dye with similar results.

Our analysis showed that SAMN-R incorporation into the cells is more biocompatible with cell motility, than is the case with metal nanoparticles [25,28] or commercial nanoparticles used in the food industry and in oral hygiene products [29].

Our results bring favorable data from SAMN-R tests on 3T3 cells and hMSCs, and from comparisons with CMFDA. However, other challenges remain for future research. All morphology, growth-activity and motility tests were made on in vitro 2D planar surfaces, whereas an evaluation of cell morphology and motility in 3D systems mimicking real tissue is needed in the next step. Furthermore, detailed monitoring of cytokine and extracellular vesicle production by MSCs and fibroblasts is required, as these cell functions are also important for effective procedures in regenerative medicine. For our recent work, we are developing an improved custom-made algorithm in MATLAB software for fully automatic cell tracking.

## 4. Materials and Methods

### 4.1. Synthesis and Characterisation of SAMN-R

Preparation of spherical-shaped SAMN-R ranging from 20 to 50 nm in size, with optimized zeta potential −22.5 mV, was based on a solid-heating method and covalent immobilization of rhodamine B isothiocyanate (RITC) on a metal surface. The method included the use of the precursor, ferric chloride FeCl_3_∙6H_2_O (Sigma-Aldrich, St. Louis, MO, USA), which was dissolved in 800 mL of deionized water. The addition of 2 g of NaBH_4_ borohydride (3.5%, 100 mL) was the next step in the procedure. The abovementioned steps were performed at 20 °C. The black precipitate was then boiled at 100 °C for two hours, cooled to room temperature, separated by an external magnet, and washed several times with water. In the final step, the intermediate product was thermally treated at 400 °C for two hours. The obtained spherical nanoparticles were dispersed in deionized water with the help of an ultrasonic bath (30 W). RITC was dissolved in 50 mM tetramethylammonium perchlorate, the solution was mixed with pure spherical nanoparticles, and the pH was adjusted to 7.0 in the presence of 50 μM RITC. After one hour of incubation, the nanoparticles were separated using an external magnetic field. A sample of bound RITC was tested by measuring absorbance at 554 nm in the supernatant. Parallel fluorospectrometric analysis was performed to confirm the RITC attachment to nanoparticles. Furthermore, the prepared SAMN-R were then diluted by sterile H_2_O (Ardeapharma, Sevetin, Czech Republic). Iron concentration was determined by an atomic absorption spectrometer (Avanta Sigma, GBC Scientific Equipment, Braeside, Australia) in acetylene-air flame. External calibration in the range of 0.5–10 mg·mL^−1^ was prepared by dilution of a certified reference material—a water calibration solution with an iron concentration of 1.000 ± 0.002 g·L^−1^ (Analytika, Prague, Czech Republic). The structure of the type of maghemite iron oxide nanoparticles and their stability were determined on the first day and one year after preparation. They were investigated by zero-field and in-field Mössbauer spectroscopy and magnetometry using a superconducting quantum interference device (SQUID, Quantum Design, San Diego, CA, USA). The distribution of nanoparticle diameter and polydispersity index in water and Dulbecco’s Modified Eagle’s Medium (DMEM, Sigma-Aldrich, St. Louis, MO, USA) were quantified by a Zetasizer Nano ZS (Malvern Instruments, Malvern, UK).

### 4.2. Cell Cultures

Two types of cells were used as a testing culture: commercial fibroblast 3T3 and MSCs isolated from patients during plastic surgery. 3T3 cell lines were obtained from Sigma-Aldrich (St. Louis, MO, USA) in a cryoconserved form. MSCs were isolated from three samples of adipose tissue from three patients (male, age 40–50, weight 80–110 kg). Adipose tissue was obtained from healthy patients undergoing abdominal liposuction (collected waste tissue) after gaining a signature of written informed consent in accordance with Czech law 372/2011 and the Declaration of Helsinki. The isolation procedure was conducted via the protocol [30].

The 3T3 cells were cultured in T25 culture dishes (Sigma-Aldrich, St. Louis, MO, USA) at 37 °C, and 5% CO_2_. High-glucose DMEM containing 10% fetal bovine serum (FBS; Sigma-Aldrich, St. Louis, MO, USA), 1% penicillin/streptomycin (Sigma-Aldrich, St. Louis, MO, USA), and 1% L-glutamine (Sigma-Aldrich, St. Louis, MO, USA) was exchanged every two days. Cells were subcultured when they reached 80 to 100% confluence using Trypsin-EDTA solution (Sigma-Aldrich, St. Louis, MO, USA).

MSCs were cultured in a 24-well plate (Sigma-Aldrich) with low glucose DMEM containing 5% FBS, 1% penicillin/streptomycin, and 1% l-glutamine. Cell passage was provided when the culture reached 80 to 100% confluence, and prewarmed Accutase (PAA Laboratories, Dartmouth, MA, USA) was applied for five minutes under 37 °C as the activator of detachment. Cells were separated from the supernatant by centrifuge for three minutes in a 1.5 mL plastic microtube (450 g).

### 4.3. Cell Labelling

When cells reached the 70 to 80% confluence level, SAMN-R in a dose of 20 µg·cm^−2^ was added to the growth medium. To study the migration of individual cells, nanoparticles were added 28 h before the start of the experiment. After 24 h, this solution was removed, cells were gently washed with phosphate buffered saline (PBS; Sigma-Aldrich, St. Louis, MO, USA) and then cultured in a growth medium without nanoparticles.

CMFDA (Thermo Fisher Scientific, Waltham, MA, USA) was used to monitor cell migration. The working solution was warmed to 37 °C before use. The growth medium was removed and the cells were gently washed by PBS. Then, the final working concentration, CMFDA of 10 µm in a serum-free medium, was added for 15 to 20 minutes, controlled by a Zeiss Axio Observer 5 fluorescence microscope (ZEISS International, Oberkochen, Germany). After this, cells were cultivated in a complete growth medium during the experiment.

Hoechst 33342 (Sigma-Aldrich, St. Louis, MO, USA) was used for nuclei staining. Dye with 10 µm working concentration was mixed with a growth medium and added to the sample. After 10 to 15 min, the solution was removed (controlled by a Zeiss Axio Observer 5 fluorescence microscope). Then, the cells were gently washed by PBS and cultured in the growth medium.

### 4.4. Confocal Microscopy

The data were acquired using a Leica TCS SP8 X confocal microscope (Leica Microsystems, Wetzlar, Germany) equipped with a pulsed white light laser (WLL), photomultiplier tubes (PMT), and a hybrid detector (HyD). The optimal SAMN-R spectrum was defined by lambda square fluorescence mapping with an excitation wavelength at 560 nm and detection range at 570 to 620 nm (maximum at 582 nm) [7].

When labelled with CMFDA, the excitation wavelength was set to 492 nm and the detection range was set between 505 and 545 nm; for the Hoechst 33342 dye, the excitation wavelength was set to 405 nm and the detection range was set between 415 and 475 nm.

Images were acquired with a spatial resolution of 1024 × 1024 pixels with a 100 Hz scan speed. The images were 1.16 × 1.16 mm in the X- and Y-axes. Following cell migration, 3D time-lapse data were obtained every five minutes over a period of six hours. Several scan fields were chosen and conducted one after another during this timeframe (mark and find function). A long-term scan was provided for use with a microscope incubator (The Stage Top Chamber, OKOLAB, Pozzuoli, Italy) with temperature and CO_2_ control.

### 4.5. Cell Morphology and Flow Cytometry

For enhanced cell adhesion, eight-well chambered cover glasses (Cellvis, Mountain View, CA, USA) were coated with a fibronectin (FN; Sigma-Aldrich, St. Louis, MO, USA) solution of 1 μg·cm^−2^ surface coverage. Then, cells were seeded in a dose of 4 × 10^3^ cells·cm^−2^ in each chamber. After three days, when the cell confluence reached 70 to 80%, the SAMN-R were added to the growth medium in each chamber with doses from 5 µg·cm^−2^ to 35 µg·cm^−2^ in increments of five, except for the control chamber. After 24 h, the medium with nanoparticles was exchanged to complete growth medium and then the cell morphology was observed using a confocal microscope.

The effect of SAMN-R labelling on stability of size, granularity, and the expression of specific cell surface ligands (CD molecules on fibroblasts and on MSCs were evaluated by a flow cytometer Cytomics FC500 (Beckman-Coulter Inc., Brea, CA, USA). The cell surface CD on fibroblasts was stained by a monoclonal antibody against CD90 conjugated to phycoerythrin (1:100 dilutions) and an antibody against CD45 conjugated to allophycocyanin (1:100 dilutions). The cell surface CD on MSCs was stained by a monoclonal antibody against CD90 conjugated to phycoerythrin (1:100 dilutions), an antibody against CD45 conjugated to allophycocyanin (1:100 dilutions), and an antibody against CD73 conjugated to fluorescein isothiocyanate (1:100 dilutions). All commercial antibodies were obtained from Miltenyi Biotec (Bergisch Gladbach, Germany). Unstained cells and cells incubated with isotype-matched controls were used to set the border value between fluorescent-positive and fluorescent-negative populations. CD positivity, cell size, and granularity of control cells and cells after nanoparticles labelling were compared (comparison of basic dot-plot forward scatter (FSC) versus side scatter (SSC)). At least 2000 cells in the non-debris gate were selected for evaluation of CD marker expression and FSC/SSC characteristic in each cell sample (MSCs labelled and unlabelled, 3T3 labelled and unlabelled), and each type of cell sample was prepared in triplicate from different patients or different in vitro cultures of cell lines.

### 4.6. Quantification of ROS Generation after SAMN-R Labelling

Reactive oxygen species (ROS) are an important factor connected with the beginning of cell stress and the development of pathological cell changes. Potential oxidative stress caused by SAMN-R was investigated by ROS analysis. hMSCs were seeded into four wells (5000 cells/well in 96-well plate) and were treated with 0, 10, 50, or 100 μg·mL^−1^ concentrations of SAMN-R. The 3T3 cells were seeded and treated in the same way. All cell samples with SAMN-R were incubated for 24 h. Afterwards, a 2 µL portion of fluorescent ROS probe (general oxidative stress indicator (CM-H2DCFDA, Thermo Fisher Scientific, Waltham, MA, USA) pre-dissolved in DMSO) was added to each well (final concentration was 10 µmol·L^−1^). The plate was placed in a CO_2_ incubator for 45 min and the fluorescent signal was measured using an Infinite PRO M200 microplate reader (Tecan, Mannedorf, Switzerland) with ex.505 nm / em.529 nm. The intensity of the fluorescence was compared with a control sample (exposed 0 μg·mL^−1^ of SAMN-R). All tests for hMSCs and 3T3 cells were repeated three times (three independent cell samples) and statistical summaries were calculated.

### 4.7. Cell Growth Curves

**Figure 10:**
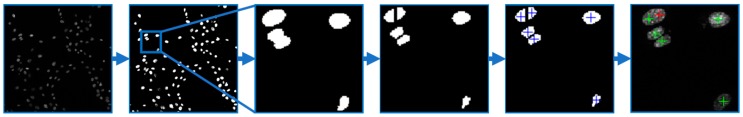
Image processing pipeline for semi-automatic cell counting. From left to right: original grey-scale image processed by a Gaussian low pass filter; thresholded binary image; resized thresholded binary image; image with nuclei divided by a watershed algorithm; image with nuclei with marked centroids; the resulting image of centroids after manual corrections mapped to the original grey-scale image.

For this experiment type, three T25 culture dishes were prepared: a control sample, SAMN-R, and CMFDA labelling. After the subculture, each cell type was seeded into nine wells in a concentration of 4 × 10^3^ cells·cm^−2^ with eight-well chambered FN-coated cover glasses. After 24, 48, and 72 h, three wells from each type were dyed with Hoechst 33342 and scanned using a confocal microscope. Each time, 10 acquired images were randomly chosen from one type of cell. The cell count was determined by counting the nuclei using a custom-made algorithm in MATLAB software (see Figure 10). The image data were filtered by a Gaussian low pass filter in order to reduce noise. Thus, the nuclei edges were enhanced due to a high value of standard deviation on the edges. The filtered image was then thresholded with an experimentally set threshold value, and the binary image of segmented cell nuclei was obtained. Due to the presence of mutually adherent cells, the nuclei were divided by a watershed algorithm using the negative parametric image, which was obtained by a distance transform applied to the binary image of the segmented nuclei. Finally, objects with a smaller area than the manually selected value were removed. For each remaining object, the centroid was found and the number of the centroids in the analyzed image was determined. The total number of the centroids corresponded to the number of cells. The program also enabled a visual evaluation of detected cells to manually correct possible false positive and/or false negative detections.

### 4.8. Analysis of Wound Healing Assay

**Figure 11:**
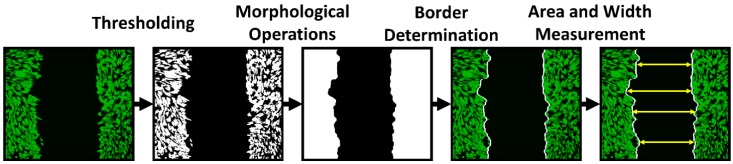
Basic pipeline of image processing approach for automatic wound healing analysis. From left to right: original grey-scale image with pseudo-colored cells; thresholded binary image; segmented image after application of morphological operations with detected scratch border; original image merged with detected scratch border; schematic of scratch width measurement.

The cells were cultured in FN-coated culture dishes (TPP Techno Plastic Products AG, Trasadingen, Switzerland) with a growth area of 9.2 cm^2^. The cells (except the cells in the control sample) were targeted with SAMN-R 24 h before the beginning of the experiment. At the beginning of the experiment, the cells had to reach 100% confluence. At first, a scratch with a p200 pipette tip was made, then the cells were gently washed with 1 mL of PBS and the solution was replaced with 3 mL of growth medium. SAMN-R-targeted and controlled cell scratches had approximately the same width. Markings were made on the outer bottom of the dish by a marker to obtain the same field of view during the image acquisition. The first images of the scratch were acquired by confocal microscope both in fluorescent and bright-field mode. The dishes were placed into the incubator at 37 °C for 24 h and images were acquired every 24 h with the same setup. Finally, the images acquired for each sample were analyzed quantitatively by a custom algorithm using MATLAB software (see Figure 11). The data acquired in the form of grey-scale fluorescence images were binarized by plain thresholding with a manually determined threshold (individually for each image). The scratch area was taken as a background (contained no cells) and the area of cells was taken as the foreground. Thus, a segmented image was treated by morphological filters, specifically by the morphological closing and filling of holes. Finally, the coordinates of the scratch border, the width of scratch in several segments, and the total scratch area were determined. This pipeline was applied for each scratch image in each time sample, and the computed parameters were statistically evaluated.

### 4.9. Analysis of Single-Cell Migration

To monitor the single hMSCs’ migration, cells were seeded onto three glass-bottom dishes (Cellvis) at a density of 1.5 × 10^3^ cells·cm^−2^. After 24 h, the experiment with a control sample was started and took six hours. At this time, one of the remaining samples was stained with CMFDA dye and the second was treated with SAMN-R. When the experiment with the control sample was finished, an experiment with a CMFDA-stained sample was conducted. A third sample was tested after 24 h of nanoparticle treatment.

In [31], we introduced our developed algorithm, created in MATLAB, for the automatic detection of CMFDA fluorescent dye-labelled cells. This method has some limitations: cells should be marked with a fluorescent dye in order to be properly detected at a low surface density. This algorithm is not suitable for non-labelled cells due to low cell detection success, so we could not use it to control samples in our experiments. For these reasons, we decided to use the manual tracking plugin by ImageJ (1.52i, National Institutes of Health, Bethesda, MD, USA) with Fiji win64 package [32,33]. Tables with cell distribution data were exported and analyzed by a custom algorithm using MATLAB software. The main parameters were accumulated distance, Euclidean distance, and velocity.

### 4.10. Statistical Analysis

Statistical analyses were performed to identify the effect of SANM-R and CMFDA labelling on cell proliferation and motility. Using the F-test, we confirmed that the data were normally distributed. Then, the paired sample *t*-test was used to test the difference between two groups: the comparison of non-labelled cells (control group) was conducted with SAMN-R-labelled cells and CMFDA-labelled cells separately. Both statistical tests were based on the determinant of the *p*-value—if *p* > 0.05, then the distinction was considered as not statistically significant. Directional statistics were provided by the Rayleigh test of uniformity—if *p* > 0.05, then the distribution of cells was considered to be homogeneous. In our case, the statistical analyses were performed using MATLAB software.

## 5. Conclusions

Fibroblasts and MSCs play key roles in the process of wound healing, as their motility and proliferation highly correlate with the speed of the regeneration of dermal scars, muscle scars, diabetic scars, infarcted scars, and other necrotic or ischemic pathology in vivo. Any probe used for staining or transfection with these two types of migratory cells should be evaluated from the perspective of biocompatibility and cytotoxicity.

Advanced microscopic methods and MATLAB image processing statistical tools allow the precise quantification of both key cell functions: motility and morphology. Our results demonstrate that the labelling process with SAMN-R with a dose lower than 20 μg·cm^−2^ does not negatively affect cell morphology, migration, cytoskeletal function, proliferation, reactive oxygen synthesis, potential for wound healing, or single-cell migration. A higher dose of SAMN-R could be a potential risk for cytoskeletal folding and could lead to detachment of the cells from the solid extracellular matrix.

## Figures and Tables

**Table 1:** Size values of the open area of narrow scratch in the wound healing assay experiment.

Item	Time	Control			SAMN-R			CMFDA		
		Min	Max	Mean	Min	Max	Mean	Min	Max	Mean
Scratch width (µm)		311.54	493.62	408.01	325.04	477.73	416.44	329.51	491.50	427.76
Scratch area (mm^2^)	0 h	0.36	0.58	0.48	0.38	0.56	0.49	0.38	0.57	0.50
	24 h	0.00	0.02	0.00	0.00	0.02	0.00	0.00	0.02	0.00

**Table 2:** Size values of the open area of wide scratch in the wound healing assay experiment.

Item	Time	Control			SAMN-R			CMFDA		
		Min	Max	Mean	Min	Max	Mean	Min	Max	Mean
Scratch width (µm)		502.18	689.57	554.24	438.18	711.15	548.73	447.23	621.20	541.37
Scratch area (mm^2^)	0 h	0.59	0.80	0.65	0.51	0.83	0.64	0.52	0.73	0.63
	24 h	0.17	0.53	0.31	0.14	0.57	0.32	0.16	0.56	0.36
	48 h	0.00	0.09	0.03	0.00	0.05	0.02	0.00	0.23	0.03

**Table 3:** Migration of hMSCs in Patient 1 from 18 cells of control, CellTracker™ Green CMFDA (5-chloromethylfluorescein diacetate) labelled, and rhodamine-derived superparamagnetic maghemite nanoparticles (SAMN-R) treatment groups. The velocity, accumulated, and Euclidean distance values for a six-hour period are shown.

	Control			SAMN-R			CMFDA		
	Min	Max	Mean	Min	Max	Mean	Min	Max	Mean
Accumulated distance (µm)	62.76	271.85	153.99	86.36	241.65	148.85	73.92	200.72	136.70
Euclidean distance (µm)	1..23	217.19	81.90	8.83	123.17	55.36	7.91	97.74	41.92
Velocity (µm/min)	0.17	0.76	0.43	0.24	0.67	0.41	0.21	0.56	0.38

**Table 4:** Migration of hMSCs in Patient 2 from 30 cells of control, CMFDA-labelled, and SAMN-R treatment groups. The velocity, accumulated, and Euclidean distance values for a six-hour period are shown.

	Control			SAMN-R			CMFDA		
	Min	Max	Mean	Min	Max	Mean	Min	Max	Mean
Accumulated distance (µm)	32.82	294.80	104.90	44.64	362.05	118.83	51.73	221.71	114.01
Euclidean distance (µm)	7.24	248.21	69.32	6.87	316.28	80.86	12.22	194.51	82.98
Velocity (µm/min)	0.09	0.82	0.29	0.12	1.01	0.33	0.14	0.62	0.32

**Table 5:** Migration of hMSCs in Patient 3 from 50 cells of control, CMFDA-labelled, and SAMN-R treatment groups. The velocity, accumulated, and Euclidean distance values for a six-hour period are shown.

	Control			SAMN-R			CMFDA		
	Min	Max	Mean	Min	Max	Mean	Min	Max	Mean
Accumulated distance (µm)	48.06	288.48	156.00	45.15	270.70	143.62	43.02	282.62	154.66
Euclidean distance (µm)	9.72	190.26	65.91	15.74	226.10	99.33	2.26	193.26	68.11
Velocity (µm/min)	0.13	0.80	0.43	0.13	0.75	0.40	0.12	0.79	0.43

**Table 6:** Migration of hMSCs in Patient 4 from 50 cells of control, CMFDA-labelled, and SAMN-R treatment groups. The velocity, accumulated, and Euclidean distance values for a six-hour period are shown.

	Control			SAMN-R			CMFDA		
	Min	Max	Mean	Min	Max	Mean	Min	Max	Mean
Accumulated distance (µm)	33.29	179.74	90.96	30.09	181.34	84.78	37.08	263.26	79.96
Euclidean distance (µm)	9.71	147.88	61.31	8.03	102.06	40.66	3.41	243.35	42.51
Velocity (µm/min)	0.09	0.50	0.25	0.08	0.50	0.24	0.10	0.73	0.22

## References

[1] Ramaswamy S., Greco J.B., Uluer M.C., Zhang Z., Zhang Z., Fishbein K.W., Spencer R.G. (2009). Magnetic Resonance Imaging of Chondrocytes Labeled with Superparamagnetic Iron Oxide Nanoparticles in Tissue-Engineered Cartilage. Tissue Eng. Part A.

[2] Sun J., Zhou S., Hou P., Yang Y., Weng J., Li X., Li M. (2007). Synthesis and Characterization of Biocompatible Fe3O4 Nanoparticles. J. Biomed. Mater. Res. Part A.

[3] Kyrtatos P.G., Lehtolainen P., Junemann-Ramirez M., Garcia-Prieto A., Price A.N., Martin J.F., Gadian D.G., Pankhurst Q.A., Lythgoe M.F. (2009). Magnetic Tagging increases Delivery of Circulating Progenitors in Vascular injury. JACC: Cardiovasc. Interv..

[4] Ito A., Hibino E., Kobayashi C., Terasaki H., Kagami H., Ueda M., Kobayashi T., Honda H. (2005). Construction and Delivery of Tissue-Engineered Human Retinal Pigment Epithelial Cell Sheets, Using Magnetite Nanoparticles and Magnetic Force. Tissue Eng..

[5] Scherer F., Anton M., Schillinger U., Henke J., Bergemann C., Krüger A., Gänsbacher B., Plank C. (2002). Magnetofection: Enhancing and Targeting Gene Delivery by Magnetic Force in vitro and in vivo. Gene Ther..

[6] Reddy L.H., Arias J.L., Nicolas J., Couvreur P. (2012). Magnetic Nanoparticles: Design and Characterization, Toxicity and Biocompatibility, Pharmaceutical and Biomedical Applications. Chem. Rev..

[7] Cmiel V., Skopalik J., Polakova K., Solar J., Havrdova M., Milde D., Justan I., Magro M., Starcuk Z., Provaznik I. (2017). Rhodamine Bound Maghemite As A Long-Term Dual Imaging Nanoprobe of Adipose Tissue-Derived Mesenchymal Stromal Cells. Eur. Biophys. J..

[8] Skopalik J., Polakova K., Havrdova M., Justan I., Magro M., Milde D., Knopfova L., Smarda J., Polakova H., Gabrielova E. (2014). Mesenchymal Stromal Cell Labeling by New Uncoated Superparamagnetic Maghemite Nanoparticles in Comparison with Commercial Resovist—An initial in vitro Study. Int. J. Nanomed..

[9] Kong B., Seog J.H., Graham L.M., Lee S.B. (2011). Experimental Considerations on the Cytotoxicity of Nanoparticles. Nanomedicine.

[10] Dai X., Liu J., Zheng H., Wichmann J., Hopfner U., Sudhop S., Prein C., Shen Y., Machens H.-G., Schilling A.F. (2017). Nano-Formulated Curcumin Accelerates Acute Wound Healing Through Dkk-1-Mediated Fibroblast Mobilization and Mcp-1-Mediated Anti-inflammation. NPG Asia Mater..

[11] Haubner F., Muschter D., Pohl F., Schreml S., Prantl L., Gassner H. (2015). A Co-Culture Model of Fibroblasts and Adipose Tissue-Derived Stem Cells Reveals New insights into Impaired Wound Healing After Radiotherapy. Int. J. Mol. Sci..

[12] Magro M., Sinigaglia G., Nodari L., Tucek J., Polakova K., Marusak Z., Cardillo S., Salviulo G., Russo U., Stevanato R. (2012). Charge Binding of Rhodamine Derivative TO Oh−Stabilized Nanomaghemite: Universal Nanocarrier For Construction of Magnetofluorescent Biosensors. Acta Biomater..

[13] Ettinger A., Wittmann T. (2014). Fluorescence Live Cell Imaging. Quantitative Imaging in Cell Biology.

[14] Jensen E.C. (2012). Use of Fluorescent Probes: Their Effect on Cell Biology and Limitations. Anat. Record.

[15] Kramer N., Walzl A., Unger C., Rosner M., Krupitza G., Hengstschläger M., Dolznig H. (2013). in vitro Cell Migration and invasion Assays. Mutat. Res./Rev. Mutat. Res..

[16] Fronza M., Heinzmann B., Hamburger M., Laufer S., Merfort I. (2009). Determination of the Wound Healing Effect of Calendula Extracts Using the Scratch Assay With 3T3 Fibroblasts. J. Ethnopharmacol..

[17] Yeom C.-H., Lee G., Park J.-H., Yu J., Park S., Yi S.-Y., Lee H., Hong Y., Yang J., Lee S. (2009). High Dose Concentration Administration of Ascorbic Acid inhibits Tumor Growth in Balb/c Mice Implanted with Sarcoma 180 Cancer Cells Via the Restriction of Angiogenesis. J. Transl. Med..

[18] Liu Q., Xu Y., Wei S., Gao W., Chen L., Zhou T., Wang Z., Ying M., Zheng Q. (2015). Mirna-148B Suppresses Hepatic Cancer Stem Cell by Targeting Neuropilin-1. Biosci. Rep..

[19] Liang C.-C., Park A.Y., Guan J.-L. (2007). in vitro Scratch Assay: A Convenient and inexpensive Method for Analysis of Cell Migration in vitro. Nat. Protoc..

[20] De Pascalis C., Etienne-Manneville S., Weaver V.M. (2017). Single and Collective Cell Migration: the Mechanics of Adhesions. Mol. Biol. Cell..

[21] Lintz M., Muñoz A., Reinhart-King C.A. (2017). the Mechanics of Single Cell and Collective Migration of Tumor Cells. J. Biomech. Eng..

[22] Jin C.-Y., Zhu B.-S., Wang X.-F., Lu Q.-H. (2008). Cytotoxicity of Titanium Dioxide Nanoparticles in Mouse Fibroblast Cells. Chem. Res. Toxicol..

[23] Coradeghini R., Gioria S., García C.P., Nativo P., Franchini F., Gilliland D., Ponti J., Rossi F. (2013). Size-Dependent Toxicity and Cell interaction Mechanisms of Gold Nanoparticles on Mouse Fibroblasts. Toxicol. Lett..

[24] Vikesland P.J., Rebodos R.L., Bottero J.Y., Rose J., Masion A. (2016). Aggregation and Sedimentation of Magnetite Nanoparticle Clusters. Environ. Sci. Nano.

[25] Pernodet N., Fang X., Sun Y., Bakhtina A., Ramakrishnan A., Sokolov J., Ulman A., Rafailovich M. (2006). Adverse Effects of Citrate/gold Nanoparticles on Human Dermal Fibroblasts. Small.

[26] Berry C.C., Wells S., Charles S., Aitchison G., Curtis A.S.G. (2004). Cell Response to Dextran-Derivatised Iron Oxide Nanoparticles Post internalisation. Biomaterials.

[27] Wu X., Tan Y., Mao H., Zhang M. (2010). Toxic Effects of Iron Oxide Nanoparticles on Human Umbilical Vein Endothelial Cells. Int. J. Nanomed..

[28] Cromer Berman S.M., Kshitiz, Wang C.J., Orukari I., Levchenko A., Bulte J.W.M., Walczak P. (2013). Cell Motility of Neural Stem Cells Is Reduced After Spio-Labeling, Which Is Mitigated After Exocytosis. Magn. Reson. Med..

[29] Tay C.Y., Cai P., Setyawati M.I., Fang W., Tan L.P., Hong C.H.L., Chen X., Leong D.T. (2013). Nanoparticles Strengthen intracellular Tension and Retard Cellular Migration. Nano Lett..

[30] Yañez R., Lamana M.L., García-Castro J., Colmenero I., Ramírez M., Bueren J.A. (2006). Adipose Tissue-Derived Mesenchymal Stem Cells Have in vivo Immunosuppressive Properties Applicable For the Control of the Graft-Versus-Host Disease. Stem Cells.

[31] Baiazitova L., Skopalik J., Cmiel V., Chmelik J., Svoboda O., Provaznik I. (2019). Modern Semi-Automatic Set-Up for Testing Cell Migration with Impact for Therapy of Myocardial infarction. World Congress on Medical Physics and Biomedical Engineering 2018.

[32] Schindelin J., Arganda-Carreras I., Frise E., Kaynig V., Longair M., Pietzsch T., Preibisch S., Rueden C., Saalfeld S., Schmid B. (2012). Fiji: An Open-Source Platform for Biological-Image Analysis. Nat. Methods.

[33] Rueden C.T., Schindelin J., Hiner M.C., DeZonia B.E., Walter A.E., Arena E.T., Eliceiri K.W. (2017). Imagej2: Imagej For the Next Generation of Scientific Image Data. BMC Bioinform..

